# Editorial: Encoding Visual Features by Parallel Ganglion Cell Initiated Pathways in the Healthy, Diseased and Artificial Retina

**DOI:** 10.3389/fncel.2019.00229

**Published:** 2019-05-24

**Authors:** Béla Völgyi, Garrett T. Kenyon, David W. Marshak, Botir Sagdullaev

**Affiliations:** ^1^Experimental Zoology and Neurobiology, University of Pécs, Pécs, Hungary; ^2^Los Alamos National Laboratory (DOE), Los Alamos, NM, United States; ^3^University of Texas Health Science Center at Houston, Houston, TX, United States; ^4^Department of Ophthalmology, Burke Neurological Institute, Weill Cornell Medicine, White Plains, NY, United States

**Keywords:** retina, ganglion cell, parallel signaling, visual coding strategies, retina model

Retinal ganglion cells (RGCs) integrate incoming signals transmitted via chemical and electrical synapses from the upstream circuitry. A combination of selective targeting by vertical retinal pathways and subtype specific computation determines the RGC encoding strategy, establishing feature selective signaling to the brain. The collective information from each RGC subtype takes shape as a feature movie (Werblin and Roska, 2007). A collection of such feature movies then are integrated by brain centers to initiate visual perception and visually guided reflexes. A mechanistic understanding of this parallel feature signaling, its pathological alterations as well as its applicability to create artificial vision is crucial on scientific, clinical, and industrial setting. Dendritic integration is one of the most essential tasks RGCs perform, thus mechanisms regulating RGC dendritic development are essential to refine dendritic size and structure. In this topical issue, Elias et al. characterized the dendritic development of JamB RGCs. While the dendritic stratification level was determined in early postnatal days (P8), fine adjustments in dendritic elongation, arbor growth, and reduction in the number of dendritic specializations take place later. Both genetic defects (knocking-out NR1 NMDARs) and suboptimal environmental conditions (light deprivation) impeded healthy maturation of the dendritic arbor indicating a role for NMDA signaling in stimulus dependent wiring in the developing mouse retina. Under optimal conditions developmental wiring results in 30 or more RGC subtypes (Völgyi et al., 2009; Baden et al., 2016), each receiving selective retinal inputs and responding to different attributes of the visual stimulus. An article by Jacoby and Schwartz reviews circuit mechanisms underlying encoding strategies of one distinctive RGC type, the suppressed-by-contrast cells (SbC). These cells decrease their maintained spiking frequency to both contrast increments and decrements, making them potent background illumination detectors. The authors further argue that SbCs form a heterogenous group providing parallel signals of illumination constancy to multiple subcortical target neuron populations.

Animal model studies gain particular importance when results are confirmed using human tissue. In this issue, Kántor et al. described the distribution of gap junction-forming connexin36 (Cx36) plaques in human RGC dendritic arbors. The authors revealed a clear tendency for Cx36 gap junctions to form clusters and to preferentially localize to terminal dendritic segments. It has been shown that certain gap junctions synchronize parasol cell spiking in primates and homologous RGCs in animal models allowing for population coding of visual features (Roy et al., 2017). The findings of Kántor et al. thus support previous descriptions in animal models and further extend those with new insights on RGC gap junction coupling. High-throughput approaches are favored methods in modern neuroscience because they yield ample data in each experiment. However, they also require quick and automatized methods to replace the tedious work of manual or half-automated data analysis. High density multi-electrode arrays allow for the examination of thousands of RGCs simultaneously. Jouty et al. presented a non-parametric, automatic scheme that uses only simple stimuli and a “spike train distance measure” as a clustering metric to achieve a quick and efficient physiological classification. By utilizing both synthetic and biological spike trains the authors show that the activity of major mouse RGC subtypes could be readily examined in a single recording session with ~1,000 cells. Moreover, given its parameter-free nature, the method is broadly applicable for the physiological classification of neurons in other structures, as well.

In retinal degenerations, photoreceptor cell loss has been shown to lead to pathway-specific changes and emergent aberrant activity across numerous RGC classes (Yee et al., 2014). Similarly, RGC morphology and function have been shown to be severely altered in the retinal tissue under stress or during pathological changes. In this issue, Lakk et al. that TRPV1 and TRPV4 expression patterns subdivide RGCs in the mouse retina into four cohorts, including: TRPV1+, TRPV4+ TRPV1/TRPV4 expressing cells and RGCs expressing neither TRP channel. The data predict that RGC subpopulations as well as the feature signals they carry will be differentially sensitive to inflammatory and mechanical stressors. In the work of Li et al. intraocular pressure elevation induced ischemia-reperfusion (IR)-related decline of ABCA1 expression. Induction of ABCA1, a protein recognized as a glaucoma risk factor, reduced RGC apoptosis and promoted anti-inflammatory factor expression, but they reduced microglial activation and pro-inflammatory cytokine expression. The authors also showed a TANK-binding kinase 1 (*TBK1*) dependent regulation of ABCA1 degradation. The results indicated a novel IR mechanism, in which TBK1-dependent ABCA1 ubiquitination leads to retinal inflammation and RGC apoptosis. Targeting the underlying signaling circuit offers a potential treatment strategy to prevent RGC apoptosis in retinal ischemia and glaucoma, two major progressive retinal conditions that eventually cause blindness. The glial S100B protein is thought to be associated with glaucoma-related RGC loss. In this issue, Kuehn et al. introduced a new model for a glaucoma-like degeneration by injecting S100B intraocularly. The injection induced a progressive degradation in RGC optic fibers that was followed by RGC degeneration and, finally, destruction of other retinal neurons. These results proved that S100B intraocular injection provides a potent model to examine the onset and progression of vision loss in glaucoma studies.

Apart from the clinical relevance, the data on parallel visual signaling streams of the retina also serves as a powerful tool in modeling and computational studies (Watkins et al., 2018). Ozimek et al. presented a biologically inspired retino-cortical mapping model that tremendously improves image analysis of Deep Convolutional Neural Nets (DCCNs). The model enables DCCNs to process large images in a single pass by utilizing only a consumer grade graphics processor (GPU), which makes it highly suitable for robot and computer vision applications.

In summary, this topical issue achieved its goal by presenting a collection of work describing mechanisms that contribute to parallel signaling, pinpointing changes that occur in pathological conditions and also showing how the comparison of artificial retinal circuits to their biological counterparts is beneficial for robotics and computer vision.

## Author Contributions

All authors listed have made a substantial, direct and intellectual contribution to the work, and approved it for publication.

### Conflict of Interest Statement

The authors declare that the research was conducted in the absence of any commercial or financial relationships that could be construed as a potential conflict of interest.
